# Model organisms contribute to diagnosis and discovery in the undiagnosed diseases network: current state and a future vision

**DOI:** 10.1186/s13023-021-01839-9

**Published:** 2021-05-07

**Authors:** Dustin Baldridge, Michael F. Wangler, Angela N. Bowman, Shinya Yamamoto, Undiagnosed Diseases Network, Maria T. Acosta, Margaret Adam, David R. Adams, Pankaj B. Agrawal, Mercedes E. Alejandro, Justin Alvey, Laura Amendola, Ashley Andrews, Euan A. Ashley, Mahshid S. Azamian, Carlos A. Bacino, Guney Bademci, Eva Baker, Ashok Balasubramanyam, Dustin Baldridge, Jim Bale, Michael Bamshad, Deborah Barbouth, Pinar Bayrak-Toydemir, Anita Beck, Alan H. Beggs, Edward Behrens, Gill Bejerano, Jimmy Bennet, Beverly Berg-Rood, Jonathan A. Bernstein, Gerard T. Berry, Anna Bican, Stephanie Bivona, Elizabeth Blue, John Bohnsack, Carsten Bonnenmann, Devon Bonner, Lorenzo Botto, Brenna Boyd, Lauren C. Briere, Elly Brokamp, Gabrielle Brown, Elizabeth A. Burke, Lindsay C. Burrage, Manish J. Butte, Peter Byers, William E. Byrd, John Carey, Olveen Carrasquillo, Ta Chen Peter Chang, Sirisak Chanprasert, Hsiao-Tuan Chao, Gary D. Clark, Terra R. Coakley, Laurel A. Cobban, Joy D. Cogan, Matthew Coggins, F. Sessions Cole, Heather A. Colley, Cynthia M. Cooper, Heidi Cope, William J. Craigen, Andrew B. Crouse, Michael Cunningham, Precilla D’Souza, Hongzheng Dai, Surendra Dasari, Joie Davis, Jyoti G. Dayal, Matthew Deardorff, Esteban C. Dell’Angelica, Shweta U. Dhar, Katrina Dipple, Daniel Doherty, Naghmeh Dorrani, Argenia L. Doss, Emilie D. Douine, David D. Draper, Laura Duncan, Dawn Earl, David J. Eckstein, Lisa T. Emrick, Christine M. Eng, Cecilia Esteves, Marni Falk, Liliana Fernandez, Carlos Ferreira, Elizabeth L. Fieg, Laurie C. Findley, Paul G. Fisher, Brent L. Fogel, Irman Forghani, Laure Fresard, William A. Gahl, Ian Glass, Bernadette Gochuico, Rena A. Godfrey, Katie Golden-Grant, Alica M. Goldman, Madison P. Goldrich, David B. Goldstein, Alana Grajewski, Catherine A. Groden, Irma Gutierrez, Sihoun Hahn, Rizwan Hamid, Neil A. Hanchard, Kelly Hassey, Nichole Hayes, Frances High, Anne Hing, Fuki M. Hisama, Ingrid A. Holm, Jason Hom, Martha Horike-Pyne, Alden Huang, Yong Huang, Laryssa Huryn, Rosario Isasi, Fariha Jamal, Gail P. Jarvik, Jeffrey Jarvik, Suman Jayadev, Lefkothea Karaviti, Jennifer Kennedy, Dana Kiley, Shilpa N. Kobren, Isaac S. Kohane, Jennefer N. Kohler, Deborah Krakow, Donna M. Krasnewich, Elijah Kravets, Susan Korrick, Mary Koziura, Joel B. Krier, Seema R. Lalani, Byron Lam, Christina Lam, Grace L. LaMoure, Brendan C. Lanpher, Ian R. Lanza, Lea Latham, Kimberly LeBlanc, Brendan H. Lee, Hane Lee, Roy Levitt, Richard A. Lewis, Sharyn A. Lincoln, Pengfei Liu, Xue Zhong Liu, Nicola Longo, Sandra K. Loo, Joseph Loscalzo, Richard L. Maas, John MacDowall, Ellen F. Macnamara, Calum A. MacRae, Valerie V. Maduro, Marta M. Majcherska, Bryan C. Mak, May Christine V. Malicdan, Laura A. Mamounas, Teri A. Manolio, Rong Mao, Kenneth Maravilla, Thomas C. Markello, Ronit Marom, Gabor Marth, Beth A. Martin, Martin G. Martin, Julian A. Martínez-Agosto, Shruti Marwaha, Jacob McCauley, Allyn McConkie-Rosell, Colleen E. McCormack, Alexa T. McCray, Elisabeth McGee, Heather Mefford, J. Lawrence Merritt, Matthew Might, Ghayda Mirzaa, Eva Morava, Paolo M. Moretti, Deborah Mosbrook-Davis, John J. Mulvihill, David R. Murdock, Anna Nagy, Mariko Nakano-Okuno, Avi Nath, Stan F. Nelson, John H. Newman, Sarah K. Nicholas, Deborah Nickerson, Shirley Nieves-Rodriguez, Donna Novacic, Devin Oglesbee, James P. Orengo, Laura Pace, Stephen Pak, J. Carl Pallais, Christina GS. Palmer, Jeanette C. Papp, Neil H. Parker, John A. Phillips, Jennifer E. Posey, Lorraine Potocki, Bradley Power, Barbara N. Pusey, Aaron Quinlan, Wendy Raskind, Archana N. Raja, Deepak A. Rao, Genecee Renteria, Chloe M. Reuter, Lynette Rives, Amy K. Robertson, Lance H. Rodan, Jill A. Rosenfeld, Natalie Rosenwasser, Francis Rossignol, Maura Ruzhnikov, Ralph Sacco, Jacinda B. Sampson, Susan L. Samson, Mario Saporta, C. Ron Scott, Judy Schaechter, Timothy Schedl, Kelly Schoch, Daryl A. Scott, Vandana Shashi, Jimann Shin, Rebecca Signer, Edwin K. Silverman, Janet S. Sinsheimer, Kathy Sisco, Edward C. Smith, Kevin S. Smith, Emily Solem, Lilianna Solnica-Krezel, Ben Solomon, Rebecca C. Spillmann, Joan M. Stoler, Jennifer A. Sullivan, Kathleen Sullivan, Angela Sun, Shirley Sutton, David A. Sweetser, Virginia Sybert, Holly K. Tabor, Amelia L. M. Tan, Queenie K.-G. Tan, Mustafa Tekin, Fred Telischi, Willa Thorson, Audrey Thurm, Cynthia J. Tifft, Camilo Toro, Alyssa A. Tran, Brianna M. Tucker, Tiina K. Urv, Adeline Vanderver, Matt Velinder, Dave Viskochil, Tiphanie P. Vogel, Colleen E. Wahl, Stephanie Wallace, Nicole M. Walley, Chris A. Walsh, Melissa Walker, Jennifer Wambach, Jijun Wan, Lee-kai Wang, Michael F. Wangler, Patricia A. Ward, Daniel Wegner, Mark Wener, Tara Wenger, Katherine Wesseling Perry, Monte Westerfield, Matthew T. Wheeler, Jordan Whitlock, Lynne A. Wolfe, Jeremy D. Woods, Shinya Yamamoto, John Yang, Muhammad Yousef, Diane B. Zastrow, Wadih Zein, Chunli Zhao, Stephan Zuchner, Tim Schedl, Stephen C. Pak, John H. Postlethwait, Jimann Shin, Lilianna Solnica-Krezel, Hugo J. Bellen, Monte Westerfield

**Affiliations:** 1 Department of Pediatrics, Washington University School of Medicine, St. Louis, MO 63110 USA; 2 Department of Molecular and Human Genetics, Baylor College of Medicine (BCM), Houston, TX 77030 USA; 3 Department of Pediatrics, BCM, Houston, TX 77030 USA; 4 Jan and Dan Duncan Neurological Research Institute, Texas Children’s Hospital, Houston, TX 77030 USA; 5 Development, Disease Models & Therapeutics Graduate Program, BCM, Houston, TX 77030 USA; 6 Department of Developmental Biology, Washington University School of Medicine, St. Louis, MO 63110 USA; 7 Center of Regenerative Medicine, Washington University in St. Louis, St. Louis, MO 63110 USA; 8 Department of Neuroscience, BCM, Houston, TX 77030 USA; 9 Department of Genetics, Washington University School of Medicine, St. Louis, MO 63110 USA; 10 Institute of Neuroscience, University of Oregon, Eugene, OR 97403 USA; 11 Howard Hughes Medical Institute, Houston, TX 77030 USA

**Keywords:** *C. elegans*, *Drosophila melanogaster*, Model organisms, Undiagnosed diseases, Zebrafish

## Abstract

Decreased sequencing costs have led to an explosion of genetic and genomic data. These data have revealed thousands of candidate human disease variants. Establishing which variants cause phenotypes and diseases, however, has remained challenging. Significant progress has been made, including advances by the National Institutes of Health (NIH)-funded Undiagnosed Diseases Network (UDN). However, 6000–13,000 additional disease genes remain to be identified. The continued discovery of rare diseases and their genetic underpinnings provides benefits to affected patients, of whom there are more than 400 million worldwide, and also advances understanding the mechanisms of more common diseases. Platforms employing model organisms enable discovery of novel gene-disease relationships, help establish variant pathogenicity, and often lead to the exploration of underlying mechanisms of pathophysiology that suggest new therapies. The Model Organism Screening Center (MOSC) of the UDN is a unique resource dedicated to utilizing informatics and functional studies in model organisms, including worm (*Caenorhabditis elegans*), fly (*Drosophila melanogaster*), and zebrafish (*Danio rerio*), to aid in diagnosis. The MOSC has directly contributed to the diagnosis of challenging cases, including multiple patients with complex, multi-organ phenotypes. In addition, the MOSC provides a framework for how basic scientists and clinicians can collaborate to drive diagnoses. Customized experimental plans take into account patient presentations, specific genes and variant(s), and appropriateness of each model organism for analysis. The MOSC also generates bioinformatic and experimental tools and reagents for the wider scientific community. Two elements of the MOSC that have been instrumental in its success are (1) multidisciplinary teams with expertise in variant bioinformatics and in human and model organism genetics, and (2) mechanisms for ongoing communication with clinical teams. Here we provide a position statement regarding the central role of model organisms for continued discovery of disease genes, and we advocate for the continuation and expansion of MOSC-type research entities as a Model Organisms Network (MON) to be funded through grant applications submitted to the NIH, family groups focused on specific rare diseases, other philanthropic organizations, industry partnerships, and other sources of support.

## The future of human genetics

Even though the human genome was sequenced in 2003, the era of functional genomics is just beginning. The deployment of next-generation sequencing revealed a staggering number of variants across individuals, with each human genome containing an average of more than 3 million single nucleotide variants when compared to the reference sequence [1, 2]. Of the approximately 20,000 human genes, only ~ 4000 are currently linked to monogenic disease and/or rare disease in Online Mendelian Inheritance in Man (OMIM) [3, 4] and Orphanet [5].

Importantly, although a single rare disease might impact only a few individuals, as a whole, rare diseases affect up to 25 million people in the US alone according to the Centers for Disease Control and Prevention (CDC) [6]. Bamshad et al. proposed that there are 6000–13,000 additional disease genes that remain to be identified for Mendelian traits and rare diseases [7]. Thus, disease gene discovery will continue for many years.

Patients with rare diseases typically have long, expensive, and frustrating diagnostic odysseys, and research with model organisms can significantly shorten their journeys by identifying causative genetic variants and disease mechanisms. The major goal of the NIH-funded MOSC, as an essential component of the UDN, is to provide experimental results to help evaluate a diagnosis, thus concluding the diagnostic odyssey. Such genetic discovery efforts typically lead to the identification of new disease genes. Although uncovering the genetic underpinnings of rare diseases for diagnosis has inherent value (e.g., for reproductive planning), it also provides significant opportunities to study rare disease biology. Such findings can contribute to a better understanding of basic biological systems and pathways, leading to development of treatments and cures and linking rare conditions with more common disease mechanisms [8, 9].

## The value of model organism screening centers

The purpose of the MOSC is to use genetic approaches in non-mammalian model organisms to evaluate the hypothesis that specific genes and variants identified in patients enrolled in the UDN are likely to cause patient clinical phenotypes. The UDN is an NIH Common Fund program arising from the earlier intramural NIH Undiagnosed Diseases Program (UDP), and now consists of a network of academic medical centers dedicated to solving medical mysteries [10]. Through the use of in-depth clinical evaluations and exome or genome sequencing and analysis, numerous patients with challenging and medically complex conditions are able to obtain a molecular diagnosis through participation in the UDN [11]. In many cases, the identification of an ‘n = 1’ potentially pathogenic variant from sequencing alone does not provide sufficient evidence that the variant is indeed causative. A subset of these cases may be solved by identifying several similarly affected patients who harbor putative pathogenic variants in the same gene, a process that is facilitated by platforms like the Matchmaker Exchange [12]. Unfortunately, this process is costly, slow, and frequently unsuccessful. Therefore, due to the recurring need for functional assessment of putative pathogenic variants, the UDN established the MOSC during Phase I of the program (September 2015 to August 2018), and expanded the MOSC in Phase II (September 2018–July 2022) [13].

The initial MOSC structure included a bioinformatics component, a *Drosophila melanogaster* (*Drosophila*; Fly) Core, and a Zebrafish Core. We note that the term “Center” is used for the overall structure of the MOSC, and the term “Core” is used for individual model organism teams due to the administrative structure specified in the NIH funding opportunity announcement. However, activities conducted by the MOSC Cores are significantly more advanced than those typically conducted by traditional research core facilities. The bioinformatics component analyzes specific genes and variants submitted, including the use of public databases of “control” individuals with respect to monogenic disease, such as ExAC and gnomAD [14, 15], and Mendelian disease databases, such as the Centers for Mendelian Genomics (CMG), to look for matching cases and variants [4, 12, 16]. These searches are integrated with specific searches throughout the literature and across model organism, gene, protein, and protein structure databases to identify tools and reagents available for potential studies in a given organism. Based on the vast amount of time spent on bioinformatic searches and the need for computational tools to help prioritize model organism studies, the Phase I MOSC developed a robust integrated platform called MARRVEL (Model organism Aggregated Resources for Rare Variant ExpLoration; http://marrvel.org/) that is freely available online and now widely used [17]. MARRVEL supports integration of more than 20 online database searches into a single search [18, 19].

**Fig. 1:**
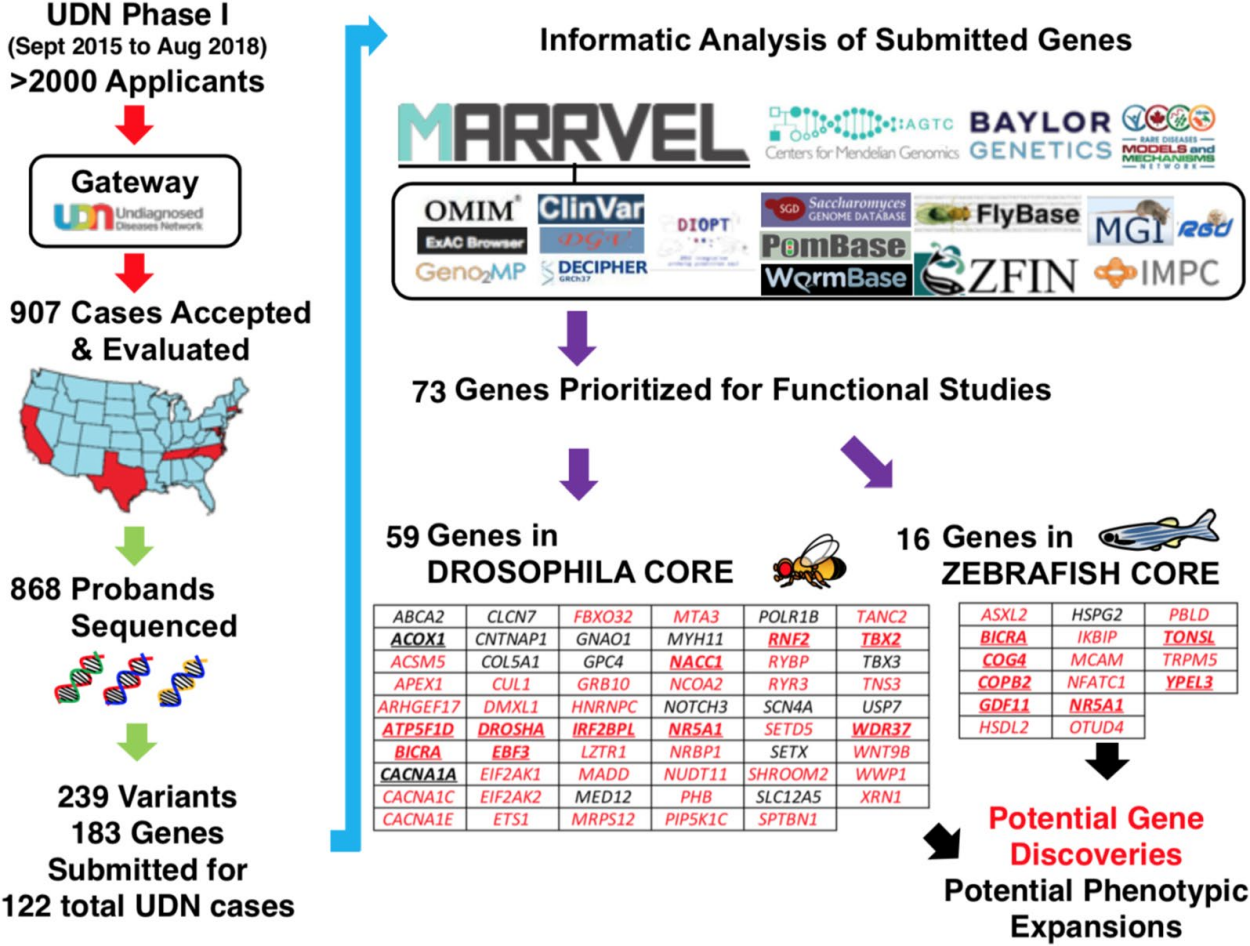
Overview of Phase I activity of the Model Organism Screening Center (MOSC) of the Undiagnosed Diseases Network (UDN). A total of 239 variants were submitted for consideration from the 907 cases evaluated at Phase I UDN Clinical Sites. States with Phase I Clinical Sites are marked in red. After bioinformatic analysis on all submissions, 59 genes were selected for study by the Fly Core and 16 genes by the Zebrafish Core. Gene names in red indicate novel disease gene candidates, whereas those in black represent proposed phenotypic expansions, according to the assessment by the clinical sites at the time of submission to the MOSC. Gene names that are in bold and underlined indicate cases where data from the MOSC directly led to a diagnosis (see Table 1 for details)

Through extensive model organism studies and the use of MARRVEL, the MOSC provided key contributions and new scientific insights during Phase I of the UDN. During this period, 239 variants in 183 genes from 122 UDN probands were submitted to the MOSC (Fig. 1). Of these, 59 genes were studied in the Fly Core and 16 in the Zebrafish Core, including two genes studied in both cores. The Phase I MOSC provided in-depth biological data for 19 genes that led directly to diagnosis (Table 1), with studies for additional genes ongoing. These discoveries included novel gene discoveries, phenotypic expansions, new biological insights, novel therapeutic targets, the ability to solve cases with only 1 or 2 patients, and extrapolation of rare undiagnosed disease mechanisms to common diseases [20].

**Table 1:** UDN MOSC diagnoses and gene discoveries

Disease gene	Disease phenotype	OMIM#	Publication
*EBF3*	Hypotonia, ataxia, and delayed development syndrome	#617330	[21]
*NACC1*	Neurodevelopmental disorder with epilepsy, cataracts, feeding difficulties, and delayed brain myelination	#617393	[22]
*CACNA1A*	Infantile developmental delay, ataxia	N/A	[23]
*ATP5F1D*	Mitochondrial complex V (ATP synthase) deficiency	#618120	[24]
*TBX2*	Vertebral anomalies and variable endocrine and T-cell dysfunction	#618223	[25]
*IRF2BPL*	Neurodevelopmental disorder with regression, abnormal movements, loss of speech, and seizures	#618088	[26]
*NR5A1*	46, XX sex reversal	#617480	[11]
*COG4*	Saul-Wilson Syndrome	#618150	[27]
*TONSL*	Spondyloepimetaphyseal dysplasia, sponastrime type	#271510	[28]
*WDR37*	Neurooculocardio-genitourinary syndrome	#618652	[29]
*ACOX1*	Schwann cell and axonal loss	#618960	[30]
TOMM70*	Hypotonia, hyperreflexia, ataxia, dystonia, and white matter abnormalities	N/A	[31]
CDK19*	Epileptic encephalopathy	#618916	[32]
*YPEL3*	Cerebral hypomyelination, abnormal peripheral nerve conduction, hypotonia, areflexia, and hypertrophic peripheral nerves	N/A	[33]
*BICRA*	Neurodevelopmental disorder with intellectual disability, autism, and dysmorphic facial features	N/A	[34]
*COPB2*	Osteoporosis, fractures, and developmental delay	N/A	[35]
*RNF2*	Intellectual disability, seizures, and dysmorphic features	N/A	[36]

Additional genes (manuscripts submitted or in preparation) include DROSHA, GDF11, MRTFB*, RAB5B*, SEC24C*, TMEM208*, and TNPO2*

^*^Cases submitted during Phase II of the UDN

The success of the Phase I MOSC led to an expansion in Phase II with an allocation of additional UDN resources to functional studies. The current MOSC incorporates a Worm (*C. elegans*) Core, a Fly (*Drosophila*) Core, and two Zebrafish Cores. The current MOSC uses a two-step evaluation system: an initial review process to screen variants primarily based on human genetics information, followed by Core level reviews to evaluate their appropriateness for specific model organism studies. As of December 2020, the Phase II MOSC has processed 143 variants in 109 genes for 108 UDN cases and assigned 60 genes for modeling in one of the three model organisms.

## MOSC discovery—historical outcomes and costs

Table 1 lists gene discoveries from the UDN MOSC in chronological order of publication and illustrates the breadth of disease phenotypes investigated. Each discovery has the potential to change medicine for that individual gene, disease, and patient and provides direct benefits outlined below. Estimating costs for each discovery is challenging due to wide variability from case to case, but based on Phase I data, an effort like the MOSC can be expected to deliver approximately six high impact gene discoveries per year for $900,000 total, or $150,000 per gene discovery. This estimate accounts for the cost associated with the discovery itself, as well as studies of other candidate disease genes for patients. Note that some efforts do not lead to diagnosis and discovery; for example, because each case typically has multiple candidate genes but typically only one is studied, failure to reveal a phenotype in a model organism may be due to study of a candidate that was not the causal gene. We note that a team-based approach increases the efficiency and lowers the cost of gene discovery through optimization of resource allocation and avoiding duplication of effort.

In addition to providing evidence that supports diagnoses, the MOSC also generates tools of significant value for further studies, such as the bioinformatic MARRVEL platform [17] and valuable in vivo reagents for the scientific community. This includes model organism mutants with loss of function alleles, lines with the patient variant(s) knocked into the endogenous gene, and tools to exogenously express human cDNA. The MOSC makes research organism reagents available to the international scientific community through NIH-supported public stock centers (*Caenorhabditis* Genetics Center, https://cgc.umn.edu; Bloomington *Drosophila* Stock Center, https://bdsc.indiana.edu; the Drosophila Genomics Research Center, dgrc.bio.indiana.edu; Zebrafish International Resource Center, https://zebrafish.org) so that they can be used for further diagnoses, in-depth mechanistic studies, and proof-of-concept translational and preclinical trial experiments.

## Benefits of undiagnosed disease gene discovery in general

Although the main goal of the UDN is to provide a diagnosis, disease gene discovery also contributes significantly to the lives of patients and their families. Gene discovery helps by: (1) ending the “diagnostic odyssey” of individual patients, reducing unnecessary diagnostic tests, offering prenatal diagnosis options for some families, and improving medical care for individual patients; (2) leading to diagnoses for patients outside of the UDN as diagnostic laboratories incorporate published new disease gene discoveries, including those from the UDN, into their sequencing interpretation and reanalysis processes; (3) facilitating the formation of social media groups, including family advocacy and support organizations that arise from the more precise molecular diagnoses; (4) enabling the future development of precision therapies that target the underlying molecular basis of rare genetic disorders and more common diseases, and (5) driving an interest in and a positive public perception of genomic research for human health, leading to greater public interest and understanding of genomics and rare and undiagnosed disease. While there is clear economic value to the patient and family members that have received a diagnosis based on functional studies performed by the MOSC, it is extremely difficult, if not impossible, to calculate the precise value of these benefits. Achieving a diagnosis prevents the added expenses for patients who would have sought evaluation from additional specialists until they get an answer, and such answers may not be found for many more years in the future if the patient’s condition is novel. In addition, the work by the MOSC has value beyond the individual UDN patient or family because the new disease gene discoveries and phenotypic expansions discovered by the MOSC (Table 1) accelerate diagnoses of patients that are not part of the UDN but who have the same genetic condition, thereby reducing costs for many families and third party payers.

## Benefits of the existing MOSC structure

**Fig. 2:**
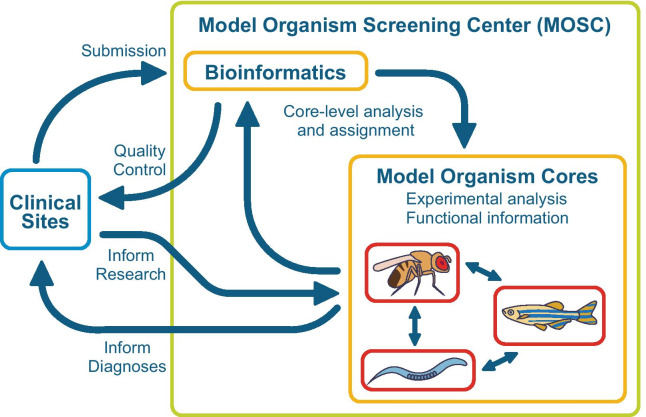
Schematic of the relationships among teams that make up the Model Organism Screening Center (MOSC). Functions of the MOSC and Clinical Sites are noted in blue. Arrows symbolize the collaborative communication among teams

The MOSC has been a productive center, and its existing structure provides an efficient mechanism for validation and further characterization of disease genes and variants using model organisms. In contrast, private companies, even those few that produce model organism reagents, do not offer model organism phenotyping. They also do not generally collaborate directly with clinicians, typically because these commercial laboratories do not have the collective expertise needed. Distributing work across model organism laboratories requires a central effort to organize and coordinate activities as well as frequent and open communication among the Model Organism Cores. For example, review of the clinical phenotype can have an impact on which model organism laboratory is best suited to study particular phenotypes or genetic pathways. The two key aspects of (1) multidisciplinary teams (Fig. 2 and Table 2) and (2) collaborative communication contribute to the high rate of gene discovery by the MOSC.

**Table 2:** Descriptions of Model Organism Screening Center (MOSC) teams

Clinical site teams	Source of candidate genes/variants; provide analysis of high-quality sequencing data in a clinical context and patient genetic and phenotypic findings
Bioinformatics team	Provides initial quality control based on human genetics, and integrates efforts from each Model Organism Core to understand evolutionarily relationships (e.g. homology and conserved synteny), known functions of candidate genes, protein structure and function, integration of information from model organism databases, availability of reagents, and previously generated knowledge of genes in models
Worm core team	Expertise in applying *C. elegans* genetics to the specific genes and variants from undiagnosed patients
Fly core team	Expertise in using *Drosophila* technology for the specific genes and variants from undiagnosed patients
Fish core team	Expertise in utilizing zebrafish genetics for the specific genes and variants from undiagnosed patients

A multidisciplinary team effort is the first and most important factor for success, because this collaboration brings together many groups spanning different areas of biological science. These benefits include: (1) bridging clinical/medical terms and model organism jargon, (2) coming to a consensus on the current understanding of the genes of interest in the context of medical genetics and model organism genetics, (3) understanding genome sequence analysis and potential pitfalls associated with DNA testing, and (4) having the unique expertise needed to develop and characterize model organism reagents that are robust and reliable to produce data relevant to the patient.

Collaboration and frequent bidirectional communication (represented by arrows in Fig. 2) is the second key feature of the MOSC. The MOSC uses a centralized system for some aspects of communication, called the UDN Gateway, which is an online system developed by the UDN Coordinating Center to facilitate data sharing and communication. The current MOSC relies on a network of expert clinical centers that are actively engaged in rare and undiagnosed diseases research, and whose participation is essential for the MOSC discovery process. Clinicians at the UDN Clinical Sites provide clinical information about the participant, explain the rationale for prioritizing candidate genes and variants that may contribute to disease phenotypes, and submit one to five genes/variants per case for further consideration. Clinical Sites submit variants to the MOSC via a built-in feature in the Gateway. Clinical sites and the MOSC teams attend a monthly call of the Model Organisms Working Group (MOWG), which facilitates communication about submissions, expected phenotypes, and model organism assignments. The MOSC also returns decisions via the Gateway to the Clinical Sites, including which model organism is appropriate for studying a specific variant, and eventually, results from model organism studies.

One of the key bidirectional communications is the interaction between Clinical Sites and the MOSC Bioinformatics Team. When Clinical Sites submit candidate variants to the Bioinformatics Team, the latter requests any additional necessary information from the Clinical Site to assess whether the gene/variant candidates are likely to be the cause of the disease before the submissions are passed on to informatics teams of each Model Organism Core. The Bioinformatics Team communicates the results of variant assessments and returns variants that are not appropriate for MOSC model organism work to the Clinical Site. The MOSC has a wide variety of genetic tools, but there are nonetheless specific variant types that are difficult to tackle using model organisms (Table 3). Currently, complex multigene interactions and environmental triggers are considered lower priority due to the scale of experimental approach that would be required to test these hypotheses. However, it is possible that new tools and resources generated in the future could be incorporated to assess these proposed mechanisms of disease in model organisms.

**Table 3:** Evaluating priorities for cases and situations that are relatively higher or lower priority for consideration by the current UDN MOSC

Higher priority cases	Lower priority cases	Situations currently beyond the scope
1. Unsolved cases 2. Novel candidate disease-causing genes 3. Potential phenotype expansions (novel variants in known disease-causing genes, but with unique phenotypes)	1. Potential complex multi-gene interactions including large copy number variants 2. Somatic or mosaic variation or risk alleles with low penetrance 3. Suspected environmental triggers 4. Potentially solved cases, including variants of uncertain significance (VUS) in known disease-causing genes with a phenotype match	1. Developing treatment or performing drug screens 2. Generating models for known genetic disorders 3. Genes that are not feasible with available tools or are cost prohibitive, such as those with no worm ortholog, human cDNA not available for fly, and duplicated genes in zebrafish

Another important set of interactions occur among the MOSC Bioinformatics Team, the Model Organism Cores, and the Clinical Sites. The information from the Clinical Sites and the bioinformatics analysis are communicated to the Cores, and in a further step, model organism experts evaluate each variant in the context of their specific model, leading to proposals for experimental work. The Bioinformatics Team and Model Organism Core teams communicate back and forth about the specific genes including homology, human genetics evidence, and hypothesized genetic mechanisms in preparation for the regular MOWG calls with the Clinical Sites. In addition, each Model Organism Core communicates directly with the other Model Organism Cores on a regular basis and during the MOWG calls, which allows the larger MOSC team as a whole to understand how each model could potentially contribute to the diagnosis of a particular undiagnosed patient. It is important to select the best model on a case-by-case basis, allowing optimization of resources for each case. Determining the Model Organism Core that is best suited to obtain diagnostic or biological insight is also part of a bidirectional dialogue involving the Clinical Sites and the MOSC Bioinformatics Team, as are discussions within and between the Model Organism Cores during the MOWG calls. Also, the cores that ultimately begin experiments on a gene communicate directly with the Clinical Site that submitted the case, so that information from the model can be conveyed to the clinicians as new data become available so that action plans can be developed.

In summary, some unique hallmarks of the MOSC are robust, bidirectional, and open communication, as well as interdisciplinary collaboration among basic scientists, human geneticists, and clinicians through regular individual meetings and monthly working group calls. This communication is an essential component of the MOSC and a key scientific justification for a MOSC structure. These multiple levels and mechanisms of communications between individuals in separate scientific fields and with complementary expertise ensure that everyone understands expectations and progress in data generation, reducing inefficiencies and potential work at cross-purposes. Beyond the UDN, the MOSC also engages members of other model organism research communities to apply the benefits of different models, dovetail efforts, and share best practices. These features could not be provided if the teams and lines of communication outlined above did not exist. In conclusion, this effort embodies a truly collaborative spirit.

## Benefits of the bioinformatic efforts of the MOSC

A robust system of informatics for quality control of potential variants is integral to MOSC operations and discoveries. In the current phase of the UDN, we have identified Human Genome Variation Society (HGVS) nomenclature issues in more than 20% of variants submitted to the MOSC. Examples include mismatch between cDNA and genomic coordinates, incorrect representations of short insertions or deletions, and mistakes when manually transcribing information from clinical genetic reports. Even though these submissions have come from top medical genetics centers, the presence of such a high error rate means that the MOSC needs a robust system to perform variant analysis and quality control. A bioinformatics team of integrated physician scientists, clinicians, bioinformaticians, rare and undiagnosed diseases researchers, geneticists, and clinical DNA testing experts facilitate this work. Providing this interdisciplinary resource for clinicians, who usually do not have model organism expertise, is a cost-effective and time-efficient mechanism for assessing the appropriateness of candidate variants for experimental analysis in each of the model systems available, discussed in detail below. The current system involves researchers at Baylor College of Medicine, Washington University in St. Louis, and the University of Oregon who analyze variants for (1) variant nomenclature, (2) minor allele frequencies in public and CMG databases, (3) gene-based metrics and prediction scores from public genomic resources, such as gnomAD, and (4) variant-based in silico prediction scores. The bioinformatics team also examines the clinical scenario as presented by the Clinical Site, studies gene information using OMIM and other databases, and confirms a shared understanding of the clinical question motivating the proposal for model organism studies. This team then communicates these data to the Model Organism Cores for further analysis, and likewise facilitates communication between the Cores and the Clinical Sites. The MARRVEL resource, discussed above, is a crucial tool designed to provide rapid access to the data needed to evaluate a candidate gene and variant for model organism studies, and has saved many hours of research time by conducting searches using this integrative tool versus separate searches across multiple databases. Bioinformatic analyses also leverage the Alliance of Genome Resources (AGR) [37], which aims to catalog human and model organism data, when reviewing model organism gene expression and functional information.

## Benefits of each model organism in the MOSC

The MOSC utilizes the experimental and genetic tools of three premier genetic model organisms: worm (*Caenorhabditis elegans*), fly (*Drosophila melanogaster*), and zebrafish (*Danio rerio*). Indeed, numerous Nobel prizes in Physiology and Medicine have been awarded to non-mammalian model organism researchers for their insights into human biology [13, 38]. Recent examples include Nobel prizes in Physiology and Medicine for circadian rhythms using fruit flies (2017), innate immunity using flies (2011), RNA interference in worm (2006), apoptosis in worm (2002), and embryonic development in flies (1995). Importantly, these are awards for contributions to medicine resulted directly from model organism studies including those organisms utilized by the MOSC.

*Caenorhabditis elegans* (*C. elegans*, a 1 mm-long nematode worm) is a major research organism for studies of animal cell and developmental biology [39]. Research in the worm has provided key insights into human biology in areas such as apoptosis, cell migration, nervous system wiring, aging, microRNAs, and insulin-like signaling, because of the conservation of molecular machines (e.g. spliceosome), intracellular pathways (e.g. autophagy), intercellular signaling pathways (e.g. Notch signaling), and multicellular processes (e.g. basement membrane biology) across animal biology [40]. The use of *C. elegans* in studies of human disease has defined new Mendelian conditions [41], uncovered phenotypic expansion [42], and provided the first key mechanistic understanding for some diseases (e.g., spinal muscular atrophy [43]). The high efficiency of knocking in patient missense variants into the orthologous *C. elegans* gene (which is uniformly done for the UDN MOSC cases), the short four-day generation time, the large body of acquired knowledge, and the publicly available biological reagents (WormBase, https://www.wormbase.org/) facilitate rapid functional studies of candidate disease gene variants. Such investigations can provide information on the pathogenicity of the patient variant, evidence in support of the mode of inheritance including the nature of dominance (e.g., antimorph vs. hypermorph), insight into disease mechanisms, and possible routes to treatment.

*Drosophila melanogaster* (fruit fly) has been used as a model organism to understand fundamental principles of genetics, developmental biology, immunity, and neuroscience for the past century [44, 45]. In the last two decades, *Drosophila* has become an important model system to dissect and understand the molecular mechanisms that underlie human diseases. This is in part because ~ 75% of human genes shown to cause human diseases were found to be conserved in *Drosophila* when the first genome-wide survey was conducted on ~ 1000 genes registered in OMIM [46]. Of the ~ 4000 human disease-linked genes currently displayed in OMIM, ~ 85% have homologs in flies. Considering that ~ 65% of protein coding genes are conserved between fly and human [17, 47], the data suggest that genes that are conserved between these species have a higher likelihood of causing genetic diseases in human. In addition to being used as a tool to dissect mechanisms of both common and rare diseases, and to explore potential therapeutic avenues, the fly has emerged as a critical tool to interpret variants of uncertain significance found in patients [20]. This is because state-of-the-art techniques to manipulate the *Drosophila* genome allow researchers to engineer flies in many different ways [48–50]. By integrating techniques to knock-out, knock-in, knock-down, or overexpress endogenous and exogenous proteins in a spatiotemporally controlled manner, fly biologists can quickly unravel the biological function of a gene of interest in vivo. One can further test whether the function of the gene is conserved between flies and human through gene-replacement experiments in which the human cDNA is used to functionally rescue loss-of-function alleles of the fly gene. In this paradigm, the ability of the human reference cDNA to rescue the fly mutations allows the testing of variants from undiagnosed patients in a relatively short (~ 6 months) time frame [44]. Detailed description and discussion of these strategies employed by the UDN MOSC fly core can be found in Bellen et al. [20]. All of this work is made possible due to rich public resources that support fly research, including a centralized database that actively collects and curates the literature (FlyBase, http://flybase.org/), public stock centers that distribute > 80,000 different strains of flies (Bloomington *Drosophila* Stock Center, https://bdsc.indiana.edu) and > 1,000,000 DNA clones (*Drosophila* Genomics Resource Center, https://dgrc.bio.indiana.edu/) supported by the NIH. Genes and variants found in an undiagnosed patient that are confirmed to be deleterious can be further studied in flies to identify disease mechanisms or test FDA-approved drugs that may be beneficial for the patient through high-throughput screens. This approach has already been effective in identifying several personalized treatments that can be returned to the bedside in a short timeframe [9, 32, 51].

The zebrafish (*Danio rerio*) has emerged as a premier organism to study human biology [52]. Being a vertebrate, zebrafish have almost all of the same organs and systems as humans, but are much smaller and develop much faster, thus supporting rapid studies at organismal, cellular, and subcellular resolution. Powerful techniques allow efficient generation, recovery, and analysis of mutations affecting genes that regulate developmental patterning, organogenesis, physiology, and behavior. It is easy to study gene function by injecting synthetic RNAs into early zebrafish embryos, generating transgenic zebrafish, or by altering gene function with genome editing technologies, such as the CRISPR/Cas9 system [53, 54]. The genome has been sequenced, and 71% of all human genes and 82% of human-disease related genes have zebrafish orthologs [55]. Targeted gene knock-out technology is robust and is the most frequent approach used by the UDN MOSC fish core, although some patient-specific knock-in models have also been generated. Further, studying zebrafish duplicates of human genes facilitates dissection of multi-function genes due to the evolutionary process of sub-functionalization that occurred after the teleost genome duplication [56, 57]. Advanced public resources facilitate these increasingly sophisticated experimental approaches in zebrafish, including a centralized database that actively collects and curates the literature (The Zebrafish Information Network, http://zfin.org) and public stock centers that distribute mutant and transgenic zebrafish strains and molecular reagents (The Zebrafish International Resource Center, https://zebrafish.org), both of which are supported by the NIH. Because organs, cell types, and gene functions are well conserved across vertebrates, analysis of zebrafish mutants provides insights into gene functions in other vertebrates, including humans [58, 59]. Zebrafish are used widely to validate candidate human disease genes and elucidate the molecular mechanisms and pathophysiology of disease [27, 28, 33, 60–62] as well as for drug discovery [63].

Often the tissue or organismal phenotype studied in worm or fly, and occasionally in zebrafish, does not resemble the phenotype of disruption of the orthologous human gene. Nevertheless, variant-induced dysfunction and genetic mechanisms can be assessed in model organisms because underlying molecular, cell biological, and genetic pathways are conserved. The term ‘phenolog’ stands for orthologous phenotypes and has been used when different phenotypes are observed from the disruption of orthologous genes [64], which occurs due to diverged organismal biology of the different species. Two examples of the use of phenologs in gene-variant assessment are wing defects in flies versus aortic abnormalities in humans, which both involve disrupted Notch signaling [65] and egg laying defects in worm versus craniosynostosis in humans caused by missense variants in *Twist* family genes [41]. The rapid assessment of the relevant phenolog for a missense variant in worms or flies provides functional information supporting a timely diagnosis. It also provides a simple phenotypic readout to dissect the underlying pathogenic genetic mechanism and supports the utility of more involved studies of cell and molecular mechanism.

The MOSC considers multiple factors when determining which model organism is most appropriate for a UDN case, including gene and variant evolutionary conservation and availability of reagents. If multiple organisms are appropriate for a single case, then the MOSC generally recommends only the simplest and fastest model organism in order to maximize the use of limited resources and to provide information to aid in a diagnosis as quickly as possible. The worm and fly lineage diverged from the human lineage before the fish and human lineages diverged, but these invertebrates allow rapid functional characterization of variants of interests and further probe into molecular mechanisms of disease. In some situations where clinical phenotypes relate to vertebrate-specific organs or cell types, zebrafish may be preferred and recommended. Another consideration is whether the proposed variant is a missense or protein truncating variant, which is straight forward for all models, or whether a patient-specific knock-in is necessary which is much more rapid in worms and flies. These decisions can be quite complex and require extensive communication among the specific Model Organism Cores and the Clinical Sites to weigh competing issues so that all parties can have a shared understanding of the organism-specific benefits and limitations of the proposed experimental work as well as the intended goal of the studies. In summary, the overall endeavor of undiagnosed disease gene discovery, the structure and multidisciplinary nature of the MOSC, and each of the Model Organism Cores all contribute to the successful diagnosis of undiagnosed patients.

## Vision for the future: proposal for a model organisms network (MON, formerly MOSC)

We propose sustaining and updating the MOSC through the creation of a Model Organisms Network (MON), which would include: (1) a central MOSC-like structure that is focused on providing functional information for timely diagnosis, and (2) deep mechanistic studies that extend to a larger network of researchers.

For (1), a MOSC-like structure, we envision continuation of a multidisciplinary central MON team, including the communication elements detailed above. We note that such an effort may extend beyond the needs or priorities of any single NIH institute or center, in keeping with the observation that most undiagnosed patients are medically complex and have multiple organ systems affected, and that undiagnosed diseases afflict both children and adults. This funding model would sustain and expand a team and system with similar concepts, structures, and components as the current MOSC, but would also integrate additional specialists in the model organism research field who have the expertise to pursue mechanistic and translational studies related to newly discovered disease genes or specific clinical phenotypes. In addition, we envision the central MON could garner additional support from philanthropy and rare disease family groups to fund mechanistic studies that not only extend and deepen discoveries from currently NIH-funded gene discovery programs like the UDN and Centers for Mendelian Genomics (CMG), but also include the many other historically identified disease genes where the underlying disease mechanism is not currently known. These mechanistic studies could focus on genes under study in the MON and on solving undiagnosed diseases.

For (2), mechanistic studies, we envision that studies by the MON would extend to examining pathways and therapeutics and constitute “deep dives” into individual genes and variants. Such studies have traditionally been funded through disparate investigator-initiated ‘R’ grant mechanisms. Although these mechanistic studies have thus far not been a formal part of the MOSC, they have been undertaken for some diseases in parallel to the ongoing diagnosis efforts using alternative funding sources, including administrative supplements, non-NIH grants, and institutional as well as philanthropic support [26, 30, 34]. We argue that the future network needs to balance ongoing disease gene discovery with deep mechanistic studies. These mechanistic studies could leverage the animal disease models and other tools generated by the MON, and could be undertaken by any external investigator with a robust approach, expertise, and reagents for investigating the gene, pathway, and disease uncovered by the MON. We suggest that these principles could establish a framework that could inform efforts beyond the current MOSC and could in principle incorporate other organisms, other funding mechanisms, and other functional approaches.

**Fig. 3:**
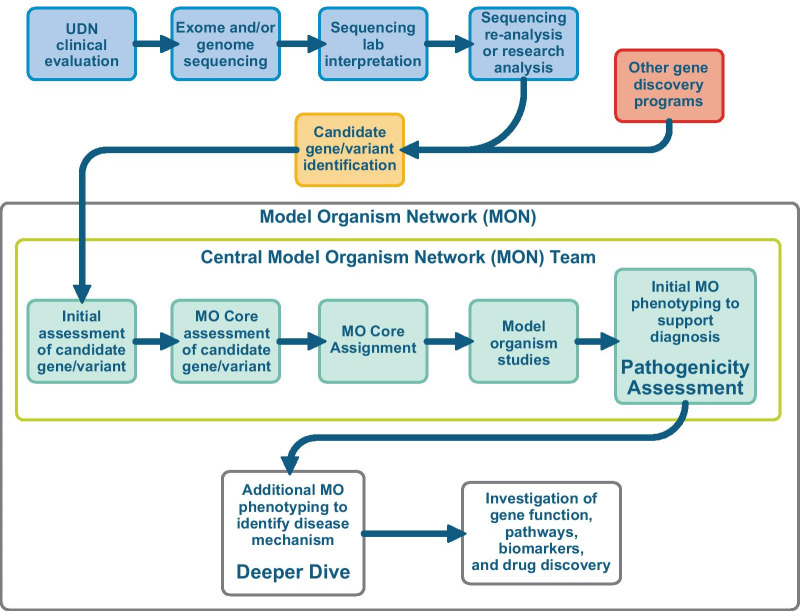
Overview of information flow and activities, including original evaluation of the patient and candidate variant identification to model organism (MO) studies. Outputs include pathogenicity assessment and, in some cases, a “deeper dive” into the underlying mechanism. Proposed Model Organism Network (MON) activities include identifying disease mechanisms for additional genes through collaborations with other model organism experts. The red box indicates potential interactions with ongoing gene discovery programs such as the Centers for Mendelian Genomics (CMG) or its future equivalents

We envision that genetic variants will continue to be submitted by clinicians in various research initiatives to the future MON and will flow through the following pipeline: (1) sequencing and bioinformatics, (2) pathogenicity studies in one of the Model Organism Cores, that includes three organism cores outlined and justified above (diagnosis), and (3) mechanistic studies in select cases (Fig. 3). The major changes we are proposing from the current MOSC workflow, and which we describe in more detail below, include the potential for expanded sources of variant submissions and the interface with deeper mechanistic studies.

## Specific components of the proposed MON

### Robust teams and communication

The future effort of the MON will require a multidisciplinary team, as well as regular communication, as exemplified by current MOWG calls and in-person meetings of the UDN. This process includes the need for a set of academic clinical centers focused on undiagnosed diseases that continue to study the most challenging cases and apply state-of-the-art genomic sequencing technologies to identify candidate variants for submission to the MON. Other needs are a MON bioinformatics team familiar with human DNA testing and sequencing data analysis to ensure quality control, and, of course, Model Organism Cores with broad biological expertise in the newest genetic technologies in each organism. Informatics efforts will become even more important because a future MON could potentially include a wider set of variant sources, leading to a greater need to harmonize data and assess each variant with consistent quality control measures.

### Variant sources from academic centers with excellence in undiagnosed diseases

In our vision of the future MON, we foresee an expansion of variant sources beyond the current UDN Clinical Sites. However, we emphasize that committed academic centers, such as the current UDN sites, are necessary to ensure successful, high quality clinical evaluations and sequencing, which are the starting points for identification of candidate disease genes and variants. We anticipate an ongoing need for timely functional studies; given the estimated 6000–13,000 additional Mendelian disease genes remaining to be identified [7] and the persistently falling costs of sequencing, patients with variants in candidate genes will continue to be identified regularly in the near future. Based on our experience, it will be necessary to have a certification process to identify sites that follow accepted practices for ensuring high quality submissions, including both clinical information and DNA sequences. We also envision that over time, sites could be educated through training modules, and that this process could lead to certification of new sites. Also, as noted above, the participation of experienced clinical teams actively engaged in identifying patients with variants in potentially novel disease genes is essential for the success of the MON. In addition to including existing UDN sites, we also propose that sources of variant submissions for MON analysis be expanded to include variants proposed by selected entities that are not presently part of the UDN. One logical choice would be for the MON to potentially collaborate with the highly successful NIH-funded CMG [66], and/or the future Mendelian Genomic Research Centers. The CMG has made more than 600 novel disease gene discoveries over the past eight years [66], and the current MOSC has already been collaborating and publishing with CMG researchers [23, 67–70]. However, an additional ~ 1200 “Tier 2” genes are not yet definitive disease genes and these cases would directly benefit from functional evaluation by the MON [66]. In addition, it may be reasonable for the MON to partner with other groups pursuing gene discovery for rare and undiagnosed diseases, including the NIH-funded Rare Diseases Clinical Research Network (RDCRN) [71], as noted below.

### MARRVEL and artificial intelligence platforms

Informatic tools provide rapid access to the data needed to evaluate a candidate gene and variant or model organism studies. The ability of computer-based methods, including artificial intelligence and deep learning, to predict the pathogenicity of variants of uncertain significance is likely to improve in coming years. The MARRVEL resource will continue to expand and add additional databases, pathogenicity prediction programs, and widgets to its platform. This type of effort is essential for the future MON. We envision that the MON will both support the development of these tools and integrate them into its workflows as they become robust, to identify appropriate candidate variants efficiently and chose the most effective model organism for variant validation.

### Model organism core teams and additional approaches

Based on the justification above and our past experience, we suggest that, at minimum, the MON will include Worm, Fly, and Fish Cores following the current structure of the MOSC. These models have proven the most successful, rapid, and cost-effective for studying undiagnosed diseases and will provide the most mechanistic insight, given the experience and increasingly sophisticated experimental tools that have been and are being developed in each system within a reasonable budget. Although the three proposed organisms have outstanding ability to model a large proportion of human variants quickly and inexpensively, cases may exist in which none of the organisms are suitable, or supplementation with human cell culture studies would provide unique information not possible with worm, fly, or fish. Based on submissions to the current MOSC, up to 10% of proposed variants in candidate human disease genes do not have sufficient evolutionary conservation to be studied in any of the three MOSC model organisms (especially when including synonymous, intronic or splicing, and UTR variants). In addition, some questions related to specific cell types affected in the patient might benefit from the use of patient biopsy or derived cells. The MON should have ways to incorporate or establish collaborations that provide mouse models, cellular transfection models, patient derived cells (e.g., fibroblasts), and human pluripotent stem cell-derived models of relevant cell or organ types whenever necessary.

The current MOSC does not take direct advantage of the mouse (*Mus musculus*) because large-scale functional studies using mice were cost-prohibitive at the time that the NIH conceived the MOSC idea (~ 2015). Considering the value of investigations using mice in the context of rare diseases [72, 73], the MOSC has been closely working with the Knockout Mouse Phenotyping Program (KOMP2, https://commonfund.nih.gov/komp2) and International Mouse Phenotyping consortium (IMPC, https://www.mousephenotype.org) to leverage the phenotypic data of null mutant animals in the informatic pipeline for variant prioritization. Due to rapid advancements in CRISPR-based gene knock-in and knock-out technologies in mouse and other species [74, 75], there is no reason for the MON to exclude any organism that can be genetically manipulated and phenotyped within a reasonable timeframe and cost.

Another complementary approach, but also beyond the current scope of the MOSC, is the use of patient-derived induced pluripotent stem cells (iPSCs), which can be differentiated into disease-relevant cell types and organoids to attempt to recapitulate the patient’s condition [76, 77]. Some current challenges inherent in the use of iPSCs include the ongoing need to develop and disseminate standardized differentiation protocols, the significant cost and time required to generate cell-types of interest, and the high degree of variability that can be observed from cell line to cell line. If highly reliable and reproducible protocols and functional assays relevant to the patient’s condition can be established with reasonable cost and timeline, such approaches will be highly synergistic with studies carried out in intact organisms, especially to test genetic variants that lack model organism orthologs and that are in human-specific non-coding elements.

## Challenges to scalability

As we describe above, much progress has been made in the development of model organism research as a tool for rare disease gene discovery. However, several challenges remain before these processes can become scalable and as easy to execute as some existing fee-for-service tests, such as exome or genome sequencing. First, disease modeling requires significant understanding of model organism biology and genetics to tailor the experimental design and analysis to the specific gene, the specific variant(s), and patient-specific clinical information in the context of the particular focal research organism. For example, to uncover a variant-specific disease mechanism, even when the null phenotype in the model organism is known, research organism experimental design often must be modified based on patient genetics, human population information, the possibility of incomplete penetrance/expressivity, and the possibility of a gain-of-function or dominant negative effect. Second, due to these complexities, the bulk of this research requires PhD-level personnel with sufficient expertise and experience to navigate the existing information, determine feasibilities of the model organism, design an experimental strategy to support pathogenicity, perform the experiments, and then bring the discovery to publication. It can be challenging to identify qualified research scientists to carry out this work in a sustainable fashion.

## Dual goals of diagnosis and mechanism

We envision NIH support for deep dives into mechanisms that would extend beyond the MON program and which would be supported by multiple NIH institutes, perhaps through competitive ‘R’ grants. Importantly, such support would also enable external model organism experts to join the MON. We are advocating for support for two distinct and important activities that will be carried out by the future MON: (1) providing rapid diagnosis and (2) uncovering disease mechanisms. To expand further, Activity (1), the diagnosis of undiagnosed diseases patients, involves using model organism experiments to provide data that solve a medical mystery for a patient in a timely manner; and Activity (2) the mechanistic understanding of previously undiagnosed diseases, includes understanding the underlying biology of disease, using rare diseases to understand common diseases, and preclinical identification and testing of therapeutics, which is a more in-depth effort.

The key feature of the components and activities of the current MOSC that distinguish it from other efforts is that they target a particular undiagnosed patient to provide timely information for diagnosis. In addition to the defining contribution of the MOSC towards diagnosis (i.e., by providing evidence for or against pathogenicity of a specific variant), the future MON should also make significant contributions towards understanding the mechanistic basis for how a variant contributes to disease pathophysiology. Although mechanistic studies are not warranted in all cases, we strongly believe that they are a powerful extension of MOSC diagnostic work on new and unstudied disease genes. Moreover, MOSC researchers generate animal models, acquire relevant expertise, and are thus well-positioned to carry out such mechanistic studies. In addition, because mechanistic studies require time, expertise, and resources, investigators outside of the central MON team should have the opportunity to drive these mechanistic studies. Given the large number of known disease genes with unknown mechanisms and expertise existing in laboratories outside of the MON, we envision that these future collaborations with experts in particular genes and pathways would become part of a larger NIH effort to uncover genetic disease mechanisms, in which the future MON might be only one of the contributors. These investigator-driven mechanistic studies could be proposed using any model organism or cellular or biochemical system, and combinations thereof. We envision that these studies could be supported by specifically targeted R01, R03, or R21 mechanisms. The Coordinating Center of the UDN has been exploring the benefit of providing funding ($150,000 per proposal) to recruit external researchers with expertise in specific genes and pathways, and these efforts have indeed facilitated the mechanistic understanding of disease mechanisms (https://undiagnosed.hms.harvard.edu/research/funding-opportunities/). The scientific justification for this dual set of goals (diagnosis and mechanism) is that the work on diagnosis must progress in a timely manner to provide answers for patients and their families. However, at the same time, more in depth biological studies, albeit slower, must also be supported to translate these discoveries to therapeutics and to common disease biology. Furthermore, even when initial studies do not support the conclusion that nominated variants cause the particular patients’ diseases, such negative results are valuable for the diagnostic mission because they prompt the clinical group to consider other candidate genes and variants. In addition, this work defines the functions of the investigated genes and variants, which might fit a different undiagnosed disease, especially for previously unstudied genes.

## Communication of the MON with other NIH-supported variant modeling efforts

Although the MOSC and future MON are unique frameworks within which to model human variants, we recognize a number of other ongoing efforts, both nationally and internationally. Some NIH-funded efforts include the Eunice Kennedy Shriver Intellectual and Developmental Disabilities Research Centers (EKS-IDDRCs, NICHD), the Rare and Atypical Diabetes Network (RADIANT, NIDDK), the Accelerating Medicines Partnership Type 2 Diabetes Consortium (AMP TD2, NIDDK), and the Rare Disease Clinical Research Network (RDCRN, NCATS). We believe these groups would benefit from ongoing and open communication with the future MON and the UDN to ensure that patients are reviewed by the most appropriate group and to avoid duplication of efforts.

## Consideration of Canadian and other international model organism approaches for rare disease

The UDN MOSC is a centralized system of several laboratories with broad expertise, knowledge, and techniques working collaboratively to solve many cases together. An alternative model is for many individual laboratories with gene-specific expertise to work on particular cases in which their genes of interest are the prime candidate of the undiagnosed condition. The Rare Diseases: Models & Mechanisms Network (RDMM) in Canada is a national network of model organism researchers and clinicians that has been using this “distributive model” of functional studies for the past four years [78]. The UDN MOSC signed a Memorandum of Understanding with the RDMM in 2016 to exchange data, knowledge, and expertise to support each other's mission.

In the RDMM approach, clinicians from around the country submit genes and variants of interest together with the clinical description of the patient. A group of clinicians that form the Clinical Advisory Committee (CAC) reviews these submissions and assesses the quality of candidate variants. The CAC passes information about appropriate gene variants to a group of biologists and bioinformaticians that form the Scientific Advisory Committee (SAC). Next, the SAC searches an internal database that contains information about model organism researchers in Canada and their expertise, to match the clinician and model organism researchers and encourage collaboration. The clinician and model organism researcher can then make specific research plans and co-submit a short research proposal back to the SAC. The SAC reviews these applications and decides whether or not to fund the project. Successful applicants receive a CAD$25,000 grant for one year to pursue the project. Interest in the project is extremely high: as of early 2020, 88% (543) of model organism laboratories across Canada had enrolled in the database, and RDMM had funded 105 projects related to 87 genes. The network published 20 peer reviewed research articles including new disease gene discoveries, phenotypic expansions of known disease genes, or mechanistic studies of known rare diseases. Due to its success in Canada, funding agencies in Japan (IRUD/J-RDMM) (https://j-rdmm.org/indexEn.html), Australia (AFGN) (https://www.functionalgenomics.org.au/), and Europe (Solve-RD) (http://solve-rd.eu/rdmm-europe/) have developed RDMM-like networks over the past two years.

Although the RDMM has been successful, there are some limitations to this model, including potential difficulties in establishing a new collaboration for each disease gene studied and the relatively limited funds and project period provided per gene. In addition, although the RDMM system is very effective in studying variants and genes for which some knowledge about their biological functions is available, genes without any in vivo studies in any pre-existing model organism tend to be left unstudied due to lack of a specific researcher with expertise. The centralized MOSC system provides flexibility and resources for researchers to tackle these “genes of unknown significance” by generating the first gene knock-out lines and other reagents. We feel that the UDN MOSC-like centralized facility that allows exploration of variants in unstudied genes with a quick turnaround time and RDMM-like matchmaking programs that involve a number of scientists and experts in well-studied genes are complementary approaches. A dual funding system such as the proposed MON that supports both types of activities will likely maximize the benefit of clinicians, basic scientists, as well as patients and family members suffering from rare and undiagnosed conditions.

## Summary and call to action

As the UDN reaches the end of its funding period from the NIH Common Fund, we propose that multiple NIH Institutes (such as NCATS, NEI, NHLBI, NHGRI, NICHD, NIDCD, NIDDK, NIGMS, NINDS, ORIP and others) work together to sustain and expand a competitive program for an ongoing UDN MOSC in the form of a MON, because most undiagnosed patients have multiple organ systems affected. It is possible that grant funding to establish the MON or MON-like structure could also be prioritized either through mechanisms such as an entirely new Common Fund initiative that is more focused on in vivo functional studies and mechanistic research, or through specific efforts by different NIH institutes. We argue that the work to sustain the MOSC and its transformation into a larger MON is highly justified and that efforts to sustain a steady pace of high impact gene discovery will pay off for rare and undiagnosed diseases, as well as impacting our understanding of common diseases.

## Data Availability

Not applicable.

## Abbreviations

AMP TD2: Accelerating Medicines Partnership Type 2 Diabetes Consortium
BCM: Baylor College of Medicine
CAC: Clinical Advisory Committee
CDC: Centers for Disease Control and Prevention
CMG: Centers for Mendelian Genomics
FFB: Foundation Fighting Blindness
HGVS: Human Genome Variation Society
NCATS: National Center for Advancing Translational Sciences
NEI: National Eye Institute
NHLBI: National Heart, Lung, and Blood Institute
NHGRI: National Human Genome Research Institute
NICHD: *Eunice Kennedy Shriver* National Institute of Child Health and Human Development
NIDCD: National Institute on Deafness and Other Communication Disorders
NIDDK: National Institute of Diabetes and Digestive and Kidney Diseases
NIGMS: National Institute of General Medical Sciences
NIH: National Institutes of Health
NINDS: National Institute of Neurological Disorders and Stroke
MARRVEL: Model organism Aggregated Resources for Rare Variant ExpLoration
MO: Model organisms
MON: Model Organisms Network
MOSC: Model Organism Screening Center
OMIM: Online Mendelian Inheritance in Man
ORIP: Office of Research Infrastructure Programs
RADIANT: Rare and Atypical Diabetes Network
RDCRN: Rare Disease Clinical Research Network
RDMM: Rare Diseases Models and Mechanisms
RFA: Request for applications
SAC: Scientific Advisory Committee
UDN: Undiagnosed Diseases Network
UDP: Undiagnosed Diseases Program
